# Effects of Positive Expressive Writing on Psychological Well-being: The Role of Locus of Control

**DOI:** 10.1192/j.eurpsy.2026.10744

**Published:** 2026-07-31

**Authors:** G. M. Festa, I. S. Lancia, A. Attouchi, F. De Pasquale, M. Manganozzi, J. Panella, L. M. Colone, M. Civino, G. Giovagnoli, C. Sarcina, M. Ciarocchi, R. Montesanto, M. E. Candia, M. Peluso

**Affiliations:** 1 Pontifical Faculty of Educational Sciences «AUXILIUM»; 2 Interdisciplinary Institute of Advanced Clinical Training «IACT», Rome, Italy

## Abstract

**Introduction:**

In recent years, an increasing body of research has focused on the effects of *positive expressive writing*, defined as the written narration of emotionally rewarding experiences, as a tool for promoting psychological well-being. This approach differs from traditional expressive writing, which focuses on traumatic or stressful events, and instead emphasizes the enhancement of positive experiences. The theoretical framework within which the present study is situated is that of ‘positive psychology’, a scientific movement, focused on the analysis of personality traits and applied methodologies that can promote psychological well-being.

**Objectives:**

This ongoing study aims to investigate the effects of positive expressive writing on specific psychological parameters (mood states), considering the moderating role of locus of control (internal vs. external).

**Methods:**

The study sample consists of 40 individuals (age: M=45, SD=6.86; gender: male=11, female=29). The experimental subjects are all individuals who report no psychological or medical conditions. They participated in the experiment voluntarily. Participants were asked to write for 20 minutes a day over three consecutive days about the most rewarding experience in their life. To measure the effects of the intervention, the Profile of Mood States (POMS) and The Locus of Control of Behavior (LCB) were administered at three time points: baseline, post-intervention (Day 3), and follow-up (Day 7). The LCB is a questionnaire designed to measure the locus of control (internal/external). The POMS assesses six mood states: Tension-Anxiety, Depression-Dejection, Anger-Hostility, Vigor-Activity, Fatigue-Inertia, and Confusion-Bewilderment. A two-way repeated measures ANOVA was conducted to analyze the interaction between locus of control type and psychological outcomes. Specific hypotheses were further explored using post-hoc LSD tests.

**Results:**

Preliminary data show that participants with an external locus of control reported a significant reduction in depression (POMS factor D) from baseline to the 7-day follow-up (13.30 → 8.17; *p* < .05). Additionally, a progressive decrease in fatigue (POMS factor F) was observed both at Day 3 (9.83 → 7.50; *p* < .05) and Day 7 (9.83 → 6.50; *p* < .005), again in the external locus group.

**Conclusions:**

Initial findings suggest that positive expressive writing may have more pronounced benefits for individuals with an external locus of control, possibly due to their greater ability to reframe positive experiences as independent of personal agency. This dispositional trait appears to enhance the intervention’s effectiveness in regulating mood and reducing fatigue. The continuation of the research will clarify whether this trend remains consistent as the sample size increases. The study, still in progress, highlights promising directions for the use of positive writing techniques in promoting psychological well-being.

**Disclosure of Interest:**

None Declared

